# Identification of Circular RNA-MicroRNA-Messenger RNA Regulatory Network in Atrial Fibrillation by Integrated Analysis

**DOI:** 10.1155/2020/8037273

**Published:** 2020-09-29

**Authors:** Tao Liu, Guoru Zhang, Yaling Wang, Mingyue Rao, Yang Zhang, Anjun Guo, Mei Wang

**Affiliations:** Department of Cardiology, The Second Hospital of Hebei Medical University, Shijiazhuang 050000, China

## Abstract

**Background:**

Circular RNA (circRNA) is a noncoding RNA that forms a closed-loop structure, and its abnormal expression may cause disease. We aimed to find potential network for circRNA-related competitive endogenous RNA (ceRNA) in atrial fibrillation (AF).

**Methods:**

The circRNA, miRNA, and mRNA expression profiles in the heart tissue from AF patients were retrieved from the Gene Expression Omnibus database and analyzed comprehensively. Differentially expressed circRNAs (DEcircRNAs), differentially expressed miRNAs (DEmiRNAs), and differentially expressed mRNAs (DEmRNAs) were identified, followed by the establishment of DEcircRNA-DEmiRNA-DEmRNA regulatory network. Functional annotation analysis of host gene of DEcircRNAs and DEmRNAs in ceRNA regulatory network was performed. In vitro experiment and electronic validation were used to validate the expression of DEcircRNAs, DEmiRNAs, and DEmRNAs.

**Results:**

A total of 1611 DEcircRNAs, 51 DEmiRNAs, and 1250 DEmRNAs were identified in AF. The DEcircRNA-DEmiRNA-DEmRNA network contained 62 circRNAs, 14 miRNAs, and 728 mRNAs. Among which, two ceRNA regulatory pairs of hsa-circRNA-100053-hsa-miR-455-5p-TRPV1 and hsa-circRNA-005843-hsa-miR-188-5p-SPON1 were identified. In addition, six miRNA-mRNA regulatory pairs including hsa-miR-34c-5p-INMT, hsa-miR-1253-DDIT4L, hsa-miR-508-5p-SMOC2, hsa-miR-943-ACTA1, hsa-miR-338-3p-WIPI1, and hsa-miR-199a-3p-RAP1GAP2 were also obtained. MTOR was a significantly enriched signaling pathway of host gene of DEcircRNAs. In addition, arrhythmogenic right ventricular cardiomyopathy, dilated cardiomyopathy, and hypertrophic cardiomyopathy were remarkably enriched signaling pathways of DEmRNAs in DEcircRNA-DEmiRNA-DEmRNA regulatory network. The expression validation of hsa-circRNA-402565, hsa-miR-34c-5p, hsa-miR-188-5p, SPON1, DDIT4L, SMOC2, and WIPI1 was consistent with the integrated analysis.

**Conclusion:**

We speculated that hsa-circRNA-100053-hsa-miR-455-5p-TRPV1 and hsa-circRNA-005843-hsa-miR-188-5p-SPON1 interaction pairs may be involved in AF.

## 1. Introduction

Atrial fibrillation (AF) is one of the most common arrhythmias and associated with heart failure [1–4]. Age, gender, obesity, and heart valve abnormalities are important factors of AF [4–6]. AF can also lead to heart failure hospitalization and death [7]. However, current treatment of AF may have adverse reactions [8, 9]. The pathogenesis of AF remains unclear. Further study of the underlying mechanisms of AF may provide new treatments for AF [10].

Circular RNAs (circRNAs) (with a covalent closed-loop structure) are considered to be the key to pathogenesis of heart disease, providing a new perspective for the pathogenesis of AF [11]. circRNA plays a crucial role in several pathophysiological processes [4, 12]. In heart disease, circRNAs function as the regulator of miRNA levels. circRNAs may be the potential biomarker. Moreover, bioinformatics analysis provides a novel perspective on circRNAs involved in AF and establishes the foundation for future research of the potential roles of circRNAs in AF [13]. miRNAs play a variety of roles in atrial fibrillation, including regulation of electrical remodeling and modulation of structural remodeling of cardiac tissue. Different miRNAs were confirmed to be up- or downregulated in AF patients [14]. Jiang et al. found the regulatory networks of has_circRNA_100612-has-miR-133b-KCNIP1/JPH2/ADRB1 and has_circRNA_405917/hsa_circRNA_008132/hsa_circRNA_104052/hsa_circRNA_101021/hsa_circRNA_101020/hsa_circRNA_102341-has-miR-892b-GJA1 in the heart tissue of AF patients [4]. However, the potential mechanism of AF remains to be studied. In this study, we performed integrated analysis based on GEO datasets to further identify dysregulated circRNAs in AF.

## 2. Methods

### 2.1. Data Collection

We obtained the expression profiles of circRNA, miRNA, and mRNA from GEO datasets by searching keywords (“Atrial fibrillation” [All Fields]) AND (“Homo sapiens” [porgn] AND “gse” [Filter]). We selected data according to the following criteria: (1) the selected dataset must be genome-wide circRNA/miRNA/mRNA transcriptome data; (2) these data were obtained from the heart tissues of the patients in the AF group and the normal control (NC) group (without drug stimulation or transfection); (3) standardized or original datasets were considered in this study. One circRNA expression dataset (GSE129409), two miRNA expression datasets (GSE68475 and GSE70887), and one mRNA expression dataset (GSE31821) were selected (Supplementary Table 1).

### 2.2. Identification of Differentially Expressed circRNAs, miRNAs, and mRNAs

Firstly, the probes corresponding to multiple circRNAs/miRNAs/mRNAs were removed. Only the single probe with the largest average expression was retained in multiple probes corresponding to circRNAs/miRNAs/mRNAs. After this treatment, qualified circRNAs/miRNAs/mRNAs were used for further analysis. Then, LIMMA package analysis was used to identify differentially expressed circRNAs and mRNAs. The metaMA package analysis was used to identify differentially expressed miRNAs. *P* value < 0.05 was the screening criteria for differentially expressed circRNAs, miRNAs, and mRNAs. Detailed data analysis process was performed as previously described [15].

### 2.3. Functional Annotation

To assess the functional annotations of host gene of DEcircRNAs and DEmRNAs in ceRNA regulatory network, Gene Ontology (GO) classification and Kyoto Encyclopedia of Genes and Genomes (KEGG) pathway enrichment analysis were conducted based on the online software GeneCodis3. Statistical significance was with the cutoff criteria of *P* value < 0.05.

### 2.4. Construction of ceRNA (DEcircRNA-DEmiRNA-DEmRNA) Regulatory Network

Firstly, starBase v3.0 was used to establish DEcircRNA-DEmiRNA regulatory network. Then, miRWalk 3.0 (http://mirwalk.umm.uni-heidelberg.de/) was utilized to find the target differentially expressed mRNAs of differentially expressed miRNAs. Finally, the DEcircRNA-DEmiRNA regulatory network was fused with the DEmiRNA-DEmRNA regulatory network to further construct the ceRNA (DEcircRNA-DEmiRNA-DEmRNA) regulatory through Cytoscape (version 3.6.1) software.

### 2.5. In Vitro Validation

The blood samples from 10 patients with AF and 10 healthy individuals were obtained for quantitative real-time polymerase chain reaction (qRT-PCR) validation. The inclusion criteria of AF patients were as follows: (1) patients were diagnosed according to the 2014 American Heart Association (AHA)/American College of Cardiology (ACC)/American Heart Rhythm Society (HRS) AF guidelines and confirmed by electrocardiogram (ECG) or Holter monitor, (2) the onset of AF in the patients was at least once a month, (3) patients were under the age of 85, (4) patients with normal liver and kidney function, and (5) patients visited the doctor at least 2 times and took medicine regularly. The exclusion criteria of AF patients were as follows: (1) patients with AF with hemodynamic disorders or malignant arrhythmia; (2) patients were 85 years old and above; (3) patients with AF with chronic cardiac insufficiency (grades II-IV); (4) patients with other severe systemic diseases, or liver and kidney failure; and (5) patients with ischemic heart disease, valvular disease, cardiomyopathy, rheumatic heart disease, primary pulmonary hypertension, and connective tissue disease. There were no statistically significant differences in age, sex, and body mass index (BMI) between AF patients and the normal individuals. We obtained the written informed consent and the approval from the ethics committee of the Second Hospital of Hebei Medical University.

Total RNA was isolated with the TRIzol reagent following the manufacturer's protocol. Based on SuperReal PreMix Plus in ABI 7500 Real-Time PCR Detection System, the qRT-PCR reactions were performed. In the 2^-*ΔΔ*Ct^ method, relative circRNA/miRNA/mRNA expression was determined. Human ACTB and GAPDH were used as endogenous controls for mRNA. In addition, human GAPDH and U6 were used as endogenous controls for circRNA and miRNA expression, respectively.

### 2.6. Electronic Validation

The GSE135445 dataset (involving 15 AF patients and 15 normal controls) was used to validate the expression of identified differentially expressed mRNAs. The result was presented as box plots. Statistical significance was ascribed to *P* value < 0.05.

## 3. Results

### 3.1. Differentially Expressed circRNAs, miRNAs, and mRNAs

**Figure 1:**
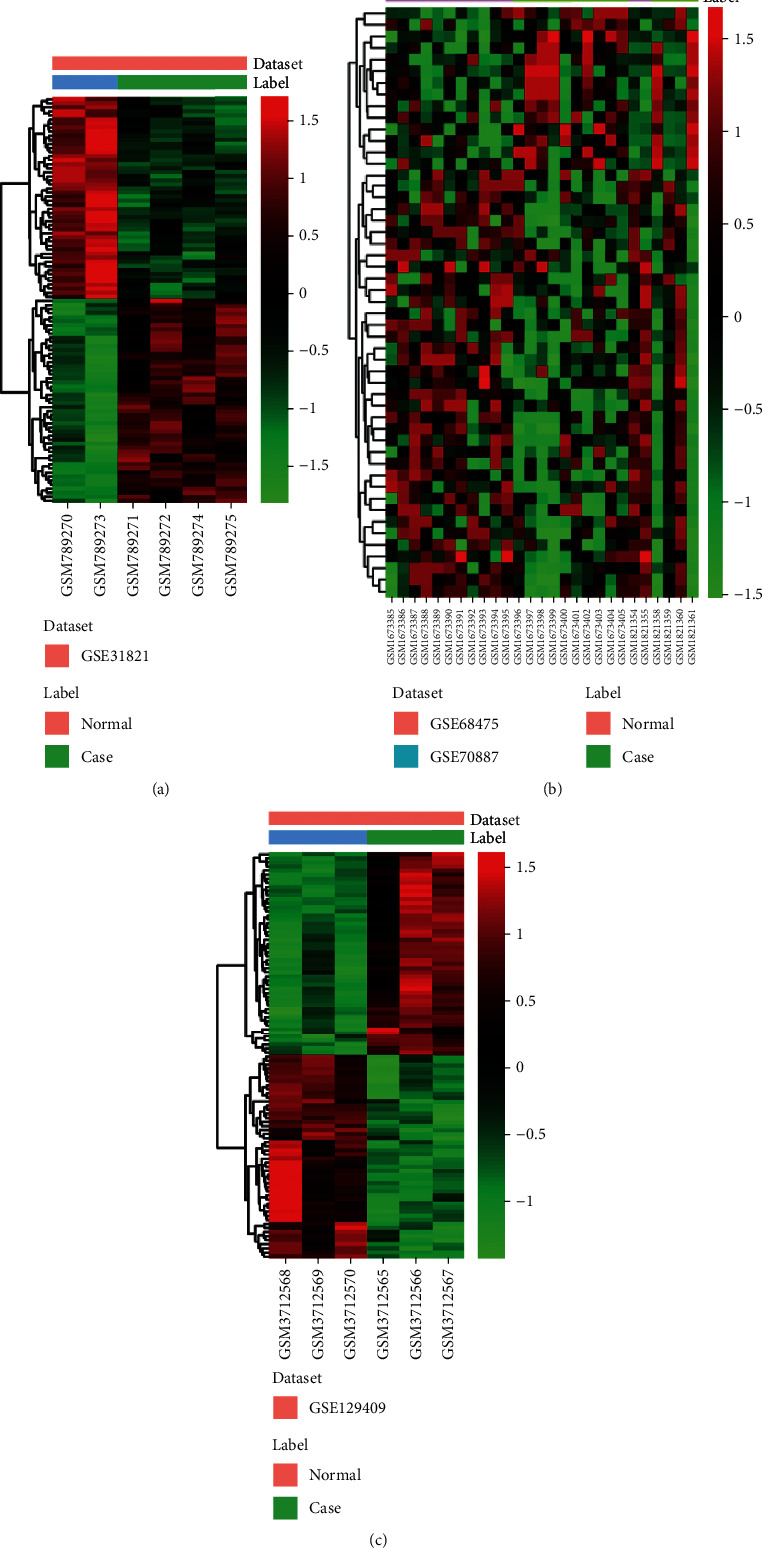
The heat map of top 100 upregulated and downregulated differentially expressed mRNAs (a), all differentially expressed miRNAs (b), and differentially expressed circRNAs (c) in AF.

Compared to normal heart tissues, a total of 1250 DEmRNAs (636 upregulated and 614 downregulated mRNAs) and 51 DEmiRNAs (14 upregulated and 38 downregulated miRNAs) were identified in AF (Figures 1(a) and 1(b)). Among them, RAP1GAP2 and ACTA1 were the most upregulated and downregulated mRNAs, respectively (Table 1); hsa-miR-508-5p and hsa-miR-99a were the most upregulated and downregulated miRNAs, respectively (Table 2). A total of 1611 DEcircRNAs (592 upregulated and 1019 downregulated circRNAs) were obtained in AF (Figure 1(c)). Among them, hsa_circRNA_405811 and hsa_circRNA_103752 were the most upregulated and downregulated circRNAs, respectively (Table 3).

### 3.2. Functional Enrichment Analysis

**Figure 2:**
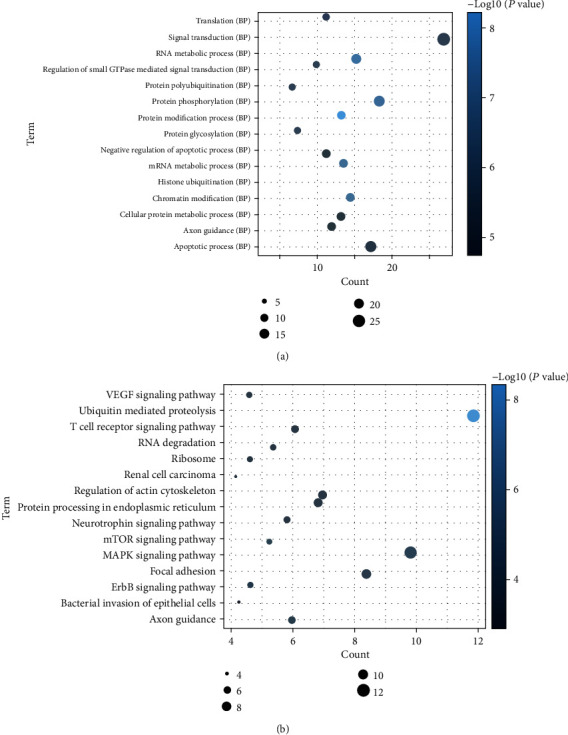
Significantly enriched biological processes and KEGG pathways of host genes of differentially expressed circRNAs. (a) BP: biological process. (b) KEGG pathways. The *x*-axis shows counts of host genes enriched in biological processes or KEGG pathways, and the *y*-axis shows biological processes or KEGG pathways.

A total of 342 host genes of DEcircRNAs were obtained. GO analysis indicated that these host genes were significantly enriched in biological processes of protein modification process (*P* = 1.99*E* − 09), RNA metabolic process (*P* = 2.57*E* − 08), and chromatin modification (*P* = 3.21*E* − 08) (Figure 2(a)). According to the KEGG pathway enrichment analysis, several pathways were identified, including ubiquitin-mediated proteolysis (*P* = 6.27*E* − 09), the mTOR signaling pathway (*P* = 0.000127701), and the MAPK signaling pathway (*P* = 0.000203168) (Figure 2(b)). In addition, significantly enriched cytological components and molecular functions of host genes are shown in Supplementary Figure 1.

### 3.3. ceRNA (DEcircRNA-DEmiRNA-DEmRNA) Regulatory Network

**Figure 3:**
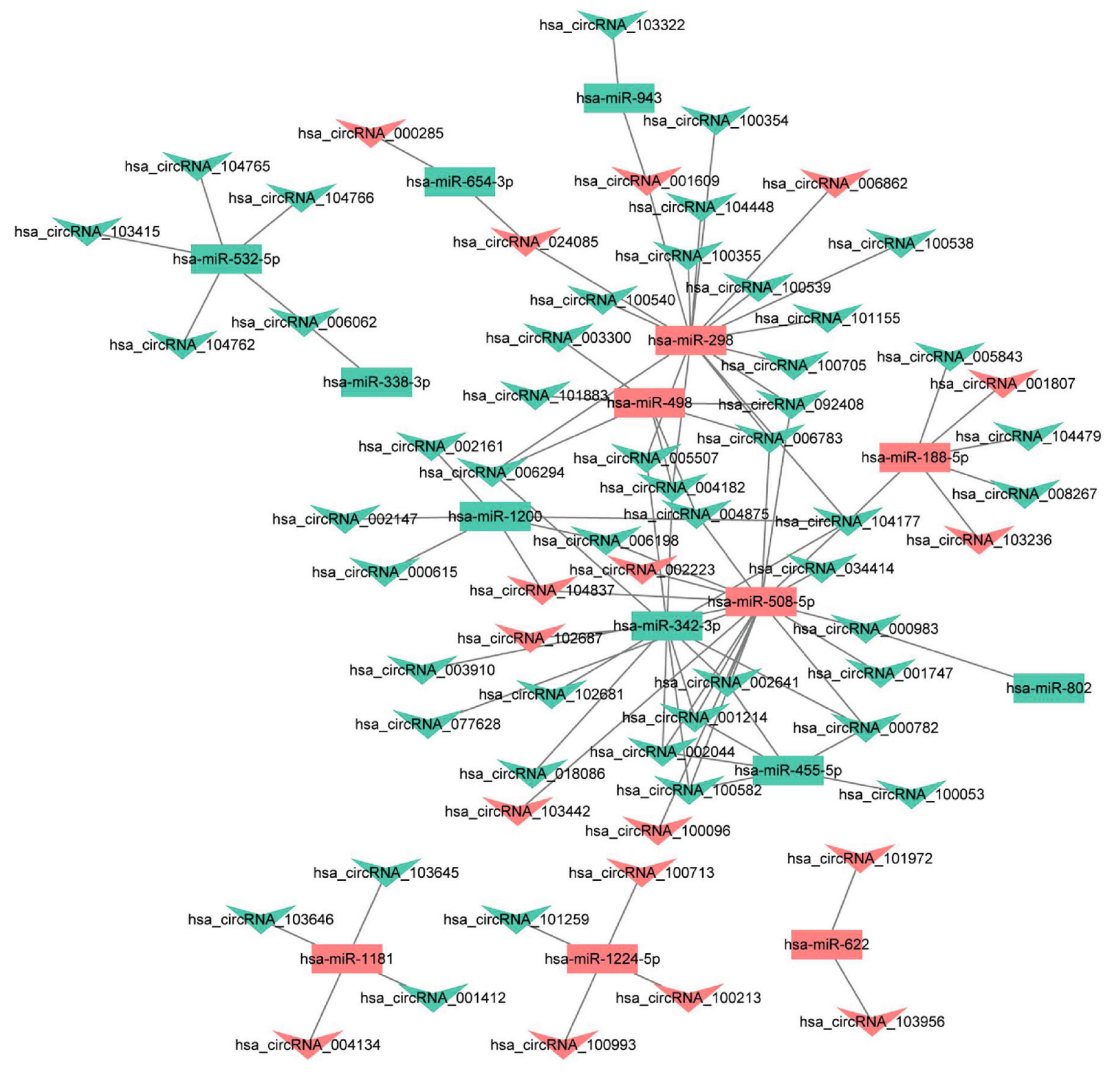
The cirRNA-miRNA network in AF. Rectangle and triangle represent circRNAs and miRNAs, respectively.

**Figure 4:**
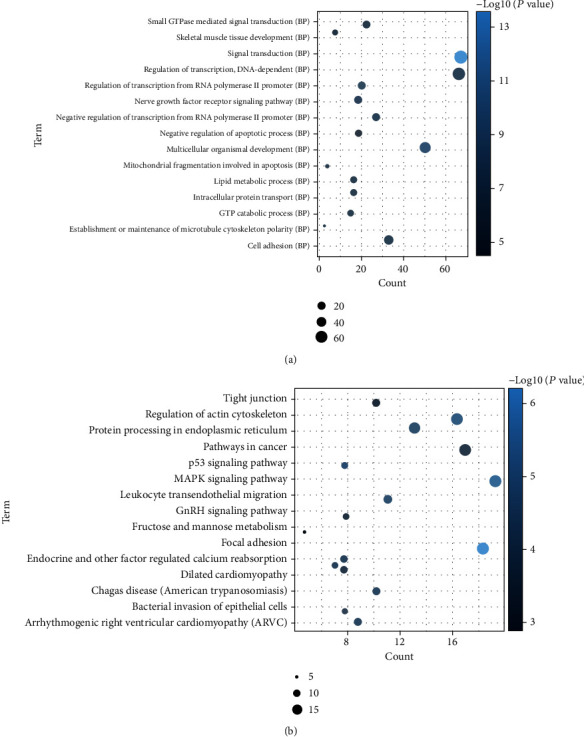
Significantly enriched biological processes and KEGG pathways of differentially expressed mRNAs in ceRNA regulatory network. (a) BP: biological process. (b) KEGG pathways. The *x*-axis shows counts of host genes enriched in biological processes or KEGG pathways, and the *y*-axis shows biological processes or KEGG pathways.

We predicted 94 DEmiRNA-DEcircRNA interactions by starBase v3.0 with a strict mode. The network was constructed by Cytoscape 3.6.1, which included 79 nodes and 94 edges (Figure 3). We also collected 32932 experimentally validated DEmiRNA-DEmRNA interactions from miRWalk 3.0 and established network by Cytoscape 3.6.1, which included 785 nodes and 2099 edges (Supplementary Figure 2). The ceRNA network contained 62 circRNAs, 14 miRNAs, and 728 mRNAs (Supplementary Figure 3). Among which, two ceRNA regulatory pairs of hsa-circRNA-100053-hsa-miR-455-5p-TRPV1 and hsa-circRNA-005843-hsa-miR-188-5p-SPON1 were identified. In addition, GO enrichment analysis revealed that these DEmRNAs were significantly enriched in biological processes of signal transduction (*P* = 7.69*E* − 14), multicellular organismal development (*P* = 3.49*E* − 09), and regulation of transcription from RNA polymerase II promoter (*P* = 1.12*E* − 07) (Figure 4(a)). According to the KEGG pathway enrichment analysis, several pathways were significantly enriched, such as arrhythmogenic right ventricular cardiomyopathy (*P* = 2.28*E* − 05), dilated cardiomyopathy (*P* = 0.000590151), and hypertrophic cardiomyopathy (*P* = 0.00173912) (Figure 4(b)). In addition, significantly enriched cytological components and molecular functions of these DEmRNAs are shown in Supplementary Figure 2.

### 3.4. qRT-PCR Validation of Selected DEmRNAs, DEmiRNAs, and DEcircRNAs

**Figure 5:**
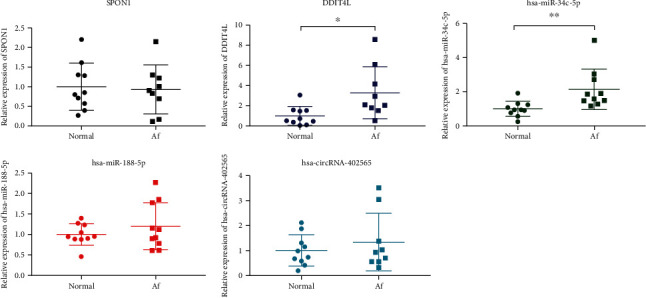
The qRT-PCR results of the DEmRNAs, DEmiRNAs, and DEcircRNAs in AF. The *x*-axis and the *y*-axis represent the group and relative expression of DEmRNAs/DEmiRNAs/DEcircRNAs, respectively. ^∗^*P* < 0.05 and ^∗∗^*P* < 0.01.

In this study, 10 patients with AF and 10 normal individuals were enrolled. Clinical information of these patients is shown in Supplementary Table 2. Two DEmRNAs (including SPON1 and DDIT4L), two DEmiRNAs (including hsa-miR-34c-5p and hsa-miR-188-5p), and one DEcircRNAs hsa-circRNA-402565 were selected randomly for qRT-PCR validation (Figure 5). DDIT4L, hsa-miR-34c-5p, hsa-miR-188-5p, and hsa-circRNA-402565 were upregulated, while SPON1 was downregulated in AF. The qRT-PCR results were in line with our integrated analysis.

### 3.5. Electronic Validation of Selected mRNAs

**Figure 6:**
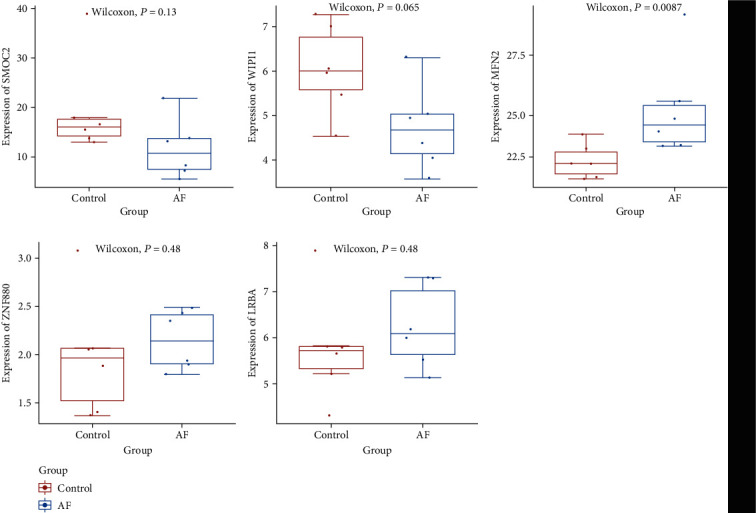
The box plots of SMOC2, WIPI1, MFN2, ZNF880, and LRBA in AF. The *x*-axis and the *y*-axis represent the group and expression of mRNAs, respectively.

In this study, two differentially expressed mRNAs (SMOC2 and WIPI1) and three host genes of circRNAs (MFN2, ZNF880, and LRBA) were randomly selected for validation (Figure 6). The result showed that SMOC2 and WIPI1 were downregulated in AF, which was consisted with our integrated analysis. The expression of MFN2, ZNF880, and LRBA was upregulated in AF. However, MFN2, ZNF880, and LRBA were not differentially expressed mRNAs in this study. Further study of MFN2, ZNF880, and LRBA in AF is needed.

## 4. Discussion

Mitofusin 2 (MFN2) is the host gene of hsa_circRNA_100053. MFN2 plays a key role in normal cardiac development [16]. MFN2 could regulate heart failure-related mitophagy by altering the mitochondrial membrane potential [17]. Chen et al. found that deletion of MFN2 leads to a spontaneous lethal dilated cardiomyopathy in mice [18]. In this study, we found that hsa_circRNA_100053 was downregulated in the heart tissue of AF patients. Moreover, downregulated hsa-miR-455-5p and target upregulated transient receptor potential vanilloid 1 (TRPV1) were under the regulation of hsa_circRNA_100053. Huang et al. reported that hsa-miR-455-5p was related to hypoxia-induced cardiomyocytes injury [19]. TRPV1 is a nonselective ion channel that preferentially obtains calcium from painful stimuli. In addition to traditional pain activation of TRPV1, TRPV1 can also be used as a universal sensor for cell damage including hypoxia. Direct activation of TRPV1 has been shown to produce cardioprotective effects on ischemia and reperfusion injury [20]. In addition, blocking TRPV1 limits the long-term preconditioning-induced cardioprotection of laparotomy [20]. An experimental study showed that TRPV1 inhibition blocked ischemic preconditioning- (IPC-) induced myocardial protection [21]. These reports suggested that hsa_circRNA_100053, hsa-miR-455-5p, and TRPV1 may play an important role in heart protection. Our study indicated that the interaction of hsa_circRNA_100053-hsa-miR-455-5p-TRPV1 may be involved in the process of AF.

Recently, there was no report about the association between hsa_circRNA_005843 and AF. Interestingly, we found that hsa_circRNA_005843 was downregulated in the heart tissue of patients with AF. Furthermore, upregulated hsa-miR-188-5p and target downregulated spondin 1 (SPON1) were regulated by hsa_circRNA_005843. hsa-miR-188-5p plays an important regulation role in the renin-angiotensin system [22]. It has been demonstrated that hsa-miR-188-5p is involved in murine cardiomyocyte biogenesis [23]. Decreased expression of hsa-miR-188-5p is found in hyperhomocysteinemia cardiomyocytes [24]. SPON1, a member of antiangiogenic family, is a sensitive plasma biomarker for early myocardial injury [25]. This indicated that hsa-miR-188-5p and SPON1 play roles in angiogenesis, which is associated with cardiomyocyte biogenesis. Our result suggested that the interaction between hsa_circRNA_005843, hsa-miR-188-5p, and SPON1 could be associated with AF.

We also found several DEmiRNA (upregulation)-DEmRNA (downregulation) regulatory pairs such as hsa-miR-34c-5p-indolethylamine N-methyltransferase (INMT), hsa-miR-1253-DNA damage-inducible transcript 4 like (DDIT4L), hsa-miR-508-5p-SPARC-related modular calcium binding 2 (SMOC2), hsa-miR-943-actin alpha 1, skeletal muscle (ACTA1), hsa-miR-338-3p-WD repeat domain, phosphoinositide-interacting 1 (WIPI1), and DEmiRNA (downregulation)-DEmRNA (upregulation) regulatory pair including hsa-miR-199a-3p-RAP1 GTPase-activating protein 2 (RAP1GAP2). Greco et al. found increased expression of hsa-miR-34c in the failing myocardium of diabetic patients [26]. INMT is differentially expressed in myocardial infarction [27]. No association between hsa-miR-1253 and AF has been reported. DDIT4L is expressed in cardiomyocytes and myocardial tissues of pathologically stressed mice, cultured neonatal rat ventricular myocyte (NRVM) models and patients with dilated cardiomyopathy. It is worth noting that pathological stress did not alter the abundance of the relevant DDIT4. DDIT4L, localizes to early endosomes, is a key regulator of NRVMs that inhibits stress-induced autophagy via mTORC1 [28]. Studies by Simonson et al. showed that cardiac pathological stress activates DDIT4L and induces autophagy by inhibiting the mTORC1 signaling pathway [28]. hsa-miR-508-5p is a potential diagnostic and prognostic marker for heart failure patients [29]. SMOC2 is involved in the inflammatory damage in the heart [30]. hsa-miR-943 is remarkably upregulated in acute ischemic stroke patients [31]. ACTA1, a contractile fiber gene, is associated with heart failure and cardiac hypertrophy [32, 33]. hsa-miR-338-3p is involved in heart failure and acute myocardial infarction [34, 35]. WIPI1 regulates mitochondrial oxidative signaling in cardiac myocytes [36]. hsa-miR-199a-3p is related to the pathophysiology of heart failure [37]. The expression of hsa-miR-199a-3p was downregulated in myocardial infarction [38]. RAP1GAP2 is associated with Chagas cardiomyopathy [39]. Thus, it can be seen that these miRNAs and target mRNAs play a key role in various cardiac pathologic processes, such as myocardial infarction, dilated cardiomyopathy, cardiac pathological stress, cardiac hypertrophy, and inflammatory damage. It is suggested that above DEmiRNAs and target DEmRNAs may be involved in the process of AF.

In addition, we found that hsa-circRNA-402565 was upregulated in the heart tissue of AF patients. Interestingly, qRT-PCR validated the expression of hsa-circRNA-402565. hsa-circRNA-402565 is downregulated in patients with ventricular septal defect [40]. It is noted that hsa-circRNA-405811 and hsa-circRNA-103752 were, respectively, the most upregulated and downregulated circRNAs in AF. hsa-miR-99a was the most downregulated miRNA in AF. Zinc finger protein 880 (ZNF880) is the host gene of hsa-circRNA-405811. The inactivating mutation of ZNF880 is found in isolated cardiac myxoma tissue samples [41]. LPS responsive beige-like anchor (LRBA) protein is the host gene of hsa-circRNA-103752. It is reported that LRBA is involved in signal transduction and vesicle trafficking in cardiogenesis [42]. The expression of LRBA is decreased 2 d after myocardial infarction [43]. It is suggested that hsa-miR-99a is involved in cardioprotective in postinfarction left ventricular remodelling [44]. Thus, it can be seen that these circRNAs involve in cardiogenesis and cardioprotection. Our result indicated that hsa-circRNA-402565, hsa-circRNA-405811, hsa-circRNA-103752, and hsa-miR-99a may be associated with the pathology of AF.

According to the functional annotation analysis, we found that mTOR was one of the most enriched signaling pathways of the host genes of DEcircRNAs. MTOR is a protein kinase that acts as an interface to a variety of metabolic pathways and is widely found in many species. MTOR plays a vital role in cellular metabolism [45]. Kinases are activated by extracellular growth factor signaling, enhancing cytoplasmic translation processes and protein synthesis. The mTOR pathway is involved in the steady-state process of the heart against stress [46]. The mTOR pathway contributes to the proliferation and survival of cardiomyocytes. In aged mice, knocking out and inhibiting mTOR can prolong survival and inhibit cardiac hypertrophy [47]. This indicated that the mTOR signaling pathway plays an important role in the heart against stress and cardiomyocyte survival, which may be associated with the development of AF.

In addition, arrhythmogenic right ventricular cardiomyopathy, dilated cardiomyopathy, and hypertrophic cardiomyopathy were three remarkably enriched signaling pathways of DEmRNAs in ceRNA regulatory network. Arrhythmogenic right ventricular cardiomyopathy is a rare inherited cardiomyopathy characterized by fibro-fatty replacement of cardiomyocytes [48, 49]. Sudden cardiac death and ventricular enlargement are the most common clinical manifestations [50]. Arrhythmogenic right ventricular cardiomyopathy mainly involves the left and right ventricle during disease progression. The enlarged left atrial is associated with the incidence of risk for death in dilated cardiomyopathy patients [51–60]. In addition, extensive atrial fibrosis is observed at autopsy in dilated cardiomyopathy patients [61]. AF is an arrhythmia often complicating the course of hypertrophic cardiomyopathy. It is found that patients with hypertrophic cardiomyopathy have a higher risk (20%) for AF [62, 63]. These reports suggested that arrhythmogenic right ventricular cardiomyopathy, dilated cardiomyopathy, and hypertrophic cardiomyopathy may be involved in the process of AF.

## 5. Conclusion

Our study found two ceRNA (DEcircRNA-DEmiRNA-DEmRNA) regulatory networks including hsa-circRNA-100053-hsa-miR-455-5p-TRPV1 and hsa-circRNA-005843-hsa-miR-188-5p-SPON1 in AF. In addition, several miRNA-mRNA regulatory pairs including hsa-miR-34c-5p-INMT, hsa-miR-1253-DDIT4L, hsa-miR-508-5p-SMOC2, hsa-miR-943-ACTA1, hsa-miR-338-3p-WIPI1, and hsa-miR-199a-3p-RAP1GAP2 and four signaling pathways such as mTOR, arrhythmogenic right ventricular cardiomyopathy, dilated cardiomyopathy, and hypertrophic cardiomyopathy were also identified. The results of the present study may provide a potential novel field into the molecular mechanisms of AF. However, there are limitations to our study. Firstly, a sample size in the qRT-PCR was small. Larger numbers of samples are further needed to validate the expression of ceRNA (DEcircRNA-DEmiRNA-DEmRNA) regulatory networks including hsa-circRNA-100053-hsa-miR-455-5p-TRPV1 and hsa-circRNA-005843-hsa-miR-188-5p-SPON1 and the most upregulated or downregulated mRNAs/miRNAs/circRNAs in AF. Secondly, he potential deeper mechanism of AF is not investigated. In vivo animal model or in vitro cell experiment is further needed to study the potential biological function of identified circRNAs, miRNAs, and mRNAs.

## Figures and Tables

**Table 1:** Top 10 upregulated and downregulated mRNAs in AF.

Symbol	Log FC	*P* value	Up/down
RAP1GAP2	0.564583	5.81*E* − 05	Up
LOC388588	0.735415	0.000103	Up
DDIT4L	0.724298	0.000187	Up
PCDHB5	0.282318	0.00063	Up
TRPV1	0.332022	0.000843	Up
LOC100509886	0.603618	0.000911	Up
C1orf93	0.370358	0.000979	Up
CCDC30	0.279824	0.001004	Up
CCL18	0.579282	0.001226	Up
MPHOSPH9	0.295236	0.001321	Up
ACTA1	-1.40916	8.30*E* − 06	Down
COLEC11	-0.68464	2.85*E* − 05	Down
SMOC2	-0.50736	8.63*E* − 05	Down
ITGB1BP3	-0.48115	0.000102	Down
INMT	-0.43338	0.00019	Down
WIPI1	-0.43174	0.000304	Down
B3GNT2	-0.6447	0.000379	Down
OSTF1	-0.32814	0.000585	Down
HEY1	-0.50453	0.0006	Down
SPON1	-0.58698	0.000735	Down

FC: fold change.

**Table 2:** Upregulated and downregulated miRNAs in AF.

ID	Combined ES	*P* value	Up/down
hsa-miR-99a	-1.948325744	1.58*E* − 05	Down
hsa-miR-199a-5p	-1.477854197	0.000457589	Down
hsa-miR-508-5p	1.418488487	0.00088982	Up
hsa-miR-199a-3p	-1.320142484	0.00135394	Down
hsa-miR-365	-1.280202941	0.002621664	Down
hsa-miR-372	1.204514016	0.003633868	Up
hsa-miR-26a-1^∗^	-1.140485814	0.004661322	Down
hsa-miR-622	1.119850033	0.005370597	Up
hsa-miR-132	1.072803396	0.007384577	Up
hsa-miR-302d	-1.047958964	0.008664093	Down
hsa-let-7f	-1.033548505	0.009485724	Down
hsa-miR-1181	1.006711572	0.011230985	Up
hsa-miR-323-3p	-0.99327606	0.012491569	Down
hsa-miR-514	-0.982260644	0.013357392	Down
hsa-miR-943	-0.97025973	0.014154981	Down
hsa-miR-328	-1.027539395	0.014920866	Down
hsa-miR-371-5p	0.96345164	0.016747391	Up
hsa-let-7i	-0.928557563	0.019493344	Down
hsa-miR-34c-5p	0.918481963	0.019551512	Up
hsa-miR-298	0.914435147	0.020064925	Up
hsa-miR-1224-5p	0.921477354	0.020188607	Up
hsa-miR-1253	-0.910171471	0.020573062	Down
hsa-let-7g	-0.914708625	0.020978097	Down
hsa-miR-660	-0.92439633	0.021108962	Down
hsa-miR-455-5p	-1.065094646	0.021971426	Down
hsa-miR-105	-0.873885194	0.025653065	Down
hsa-miR-7-2_∗_	-0.870938842	0.026112318	Down
hsa-miR-342-3p	-0.914017107	0.029399291	Down
hsa-miR-412	-0.848482991	0.029880717	Down
hsa-miR-345	0.847634702	0.030183762	Up
hsa-miR-188-5p	0.84774053	0.03099467	Up
hsa-miR-532-5p	-0.847445391	0.032511465	Down
hsa-miR-1296	-0.83188883	0.033097822	Down
hsa-miR-150	-0.92357357	0.034421637	Down
hsa-miR-551b	-0.821543289	0.035474621	Down
hsa-miR-181a-2^∗^	-0.816558501	0.036074016	Down
hsa-miR-338-3p	-0.822800891	0.03637736	Down
hsa-miR-654-3p	-0.810794989	0.037860622	Down
hsa-miR-498	0.806871169	0.038377453	Up
hsa-miR-944	-0.801311236	0.039480709	Down
hsa-miR-208b	0.79839323	0.040113362	Up
hsa-miR-16-2^∗^	-0.824929773	0.040444012	Down
hsa-miR-143	-0.791385474	0.042083126	Down
hsa-miR-597	-0.789967814	0.04214698	Down
hsa-miR-433	-0.788037698	0.042547929	Down
hsa-miR-24-2^∗^	-0.782812124	0.043926008	Down
hsa-miR-450a	-0.781978746	0.044964884	Down
hsa-miR-802	-0.776547306	0.045503217	Down
hsa-miR-1200	-0.76825428	0.047587947	Down
hsa-miR-513c	0.767204868	0.04833756	Up
hsa-miR-891a	-0.760813039	0.049622889	Down

ES: effect size. The bigger the absolute value of combined ES, the bigger the fold change.

**Table 3:** Top 10 upregulated and downregulated circRNAs in AF.

ID	Alias	Log FC	*P* value	Up/down
hsa_circRNA_405811		1.910908	5.86*E* − 05	Up
hsa_circRNA_058161	hsa_circ_0058161	1.625918	0.000148	Up
hsa_circRNA_100693	hsa_circ_0020174	1.472556	0.00026	Up
hsa_circRNA_404814		1.485693	0.000344	Up
hsa_circRNA_102950	hsa_circ_0058794	1.575402	0.000409	Up
hsa_circRNA_102949	hsa_circ_0058792	1.48237	0.000424	Up
hsa_circRNA_001873	hsa_circ_0001873	1.394786	0.000522	Up
hsa_circRNA_100372	hsa_circ_0015004	1.350013	0.000533	Up
hsa_circRNA_402565		1.299606	0.000622	Up
hsa_circRNA_406752		1.394682	0.00064	Up
hsa_circRNA_103752	hsa_circ_0006867	-1.27376	0.000302	Down
hsa_circRNA_102831	hsa_circ_0001074	-1.41386	0.000534	Down
hsa_circRNA_104315	hsa_circ_0079480	-1.49241	0.000718	Down
hsa_circRNA_079477	hsa_circ_0079477	-1.28623	0.000724	Down
hsa_circRNA_005791	hsa_circ_0005791	-1.7986	0.001027	Down
hsa_circRNA_103416	hsa_circ_0005299	-1.87406	0.001123	Down
hsa_circRNA_104750	hsa_circ_0008678	-1.07604	0.001253	Down
hsa_circRNA_004825	hsa_circ_0004825	-1.08205	0.001293	Down
hsa_circRNA_004491	hsa_circ_0004491	-1.17035	0.001311	Down
hsa_circRNA_406303		-1.30032	0.001433	Down

FC: fold change.

## Data Availability

All data are available in the article.

## Supplementary Materials

Supplementary 1 Supplementary Figure 1:significantly enriched cytological components and molecular functions of host genes of differentially expressed circRNAs. (A) CC, cytological components; (B) MF, molecular functions. The *x*-axis shows counts of host genes enriched in cytological components or molecular functions and the *y*-axis shows biological processes or molecular functions.

Supplementary 2 Supplementary Figure 2: the miRNA-mRNA network in AF. Rectangle and ellipse represent miRNAs and mRNA, respectively. Red and green colors represent upregulation and downregulation, respectively.

Supplementary 3 Supplementary Figure 3: ceRNA (DEcircRNA-DEmiRNA-DEmRNA) regulatory network. The trigonal nodes, rectangle nodes, and elliptical nodes indicate DEcircRNAs, DEmiRNAs, and DEmRNAs, respectively. Red and green colors represent upregulation and downregulation, respectively. Nodes with the black border were DEcircRNA/DEmiRNA/DEmRNA derived from the top 10 upregulated and downregulated DEcircRNA/DEmiRNA/DEmRNA in AF.

Supplementary 4 Supplementary Figure 4: significantly enriched cytological components and molecular functions of differentially expressed mRNAs in ceRNA regulatory network. (A) CC, cytological components; (B) MF, molecular functions. The *x*-axis shows counts of host genes enriched in cytological components or molecular functions, and the *y*-axis shows biological processes or molecular functions.

Supplementary 5 Supplementary Table 2: the clinical information of AF patients and normal individuals in the qRT-PCR.

Supplementary 6 Supplementary Table 1: selected circRNA, miRNA, and mRNA datasets.
