# Supplementary figures and images for: Identification of Circular RNA-MicroRNA-Messenger RNA Regulatory Network in Atrial Fibrillation by Integrated Analysis
Source: Biomed Res Int. 2020 Sep 29;2020:8037273. doi: 10.1155/2020/8037273 (PMC7545447; doi:10.1155/2020/8037273)

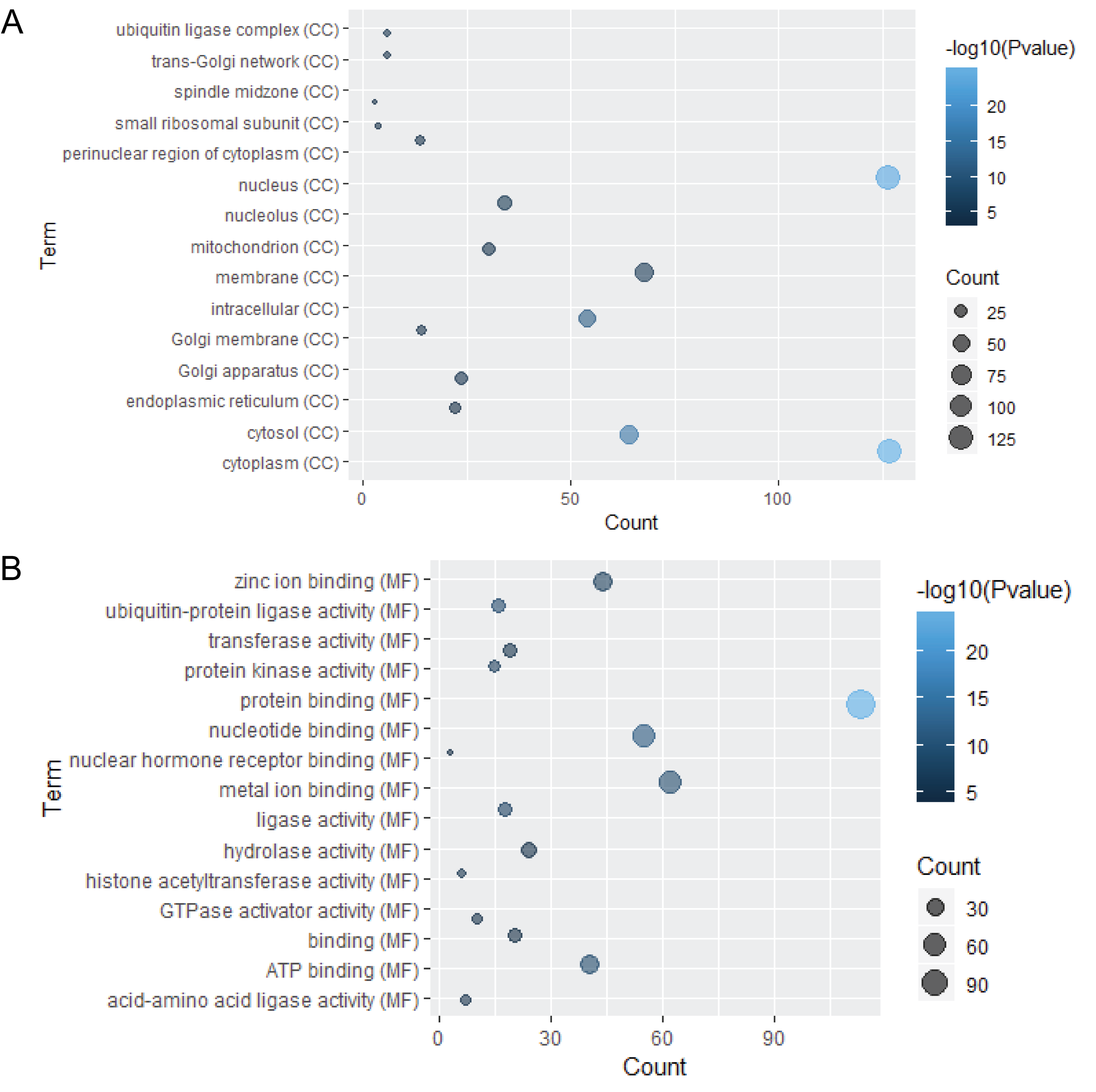

Supplement: Supplementary 1 — Supplementary Figure 1:significantly enriched cytological components and molecular functions of host genes of differentially expressed circRNAs. (A) CC, cytological components; (B) MF, molecular functions. The x-axis shows counts of host genes enriched in cytological components or molecular functions and the y-axis shows biological processes or molecular functions. [file 8037273.f1.tif]

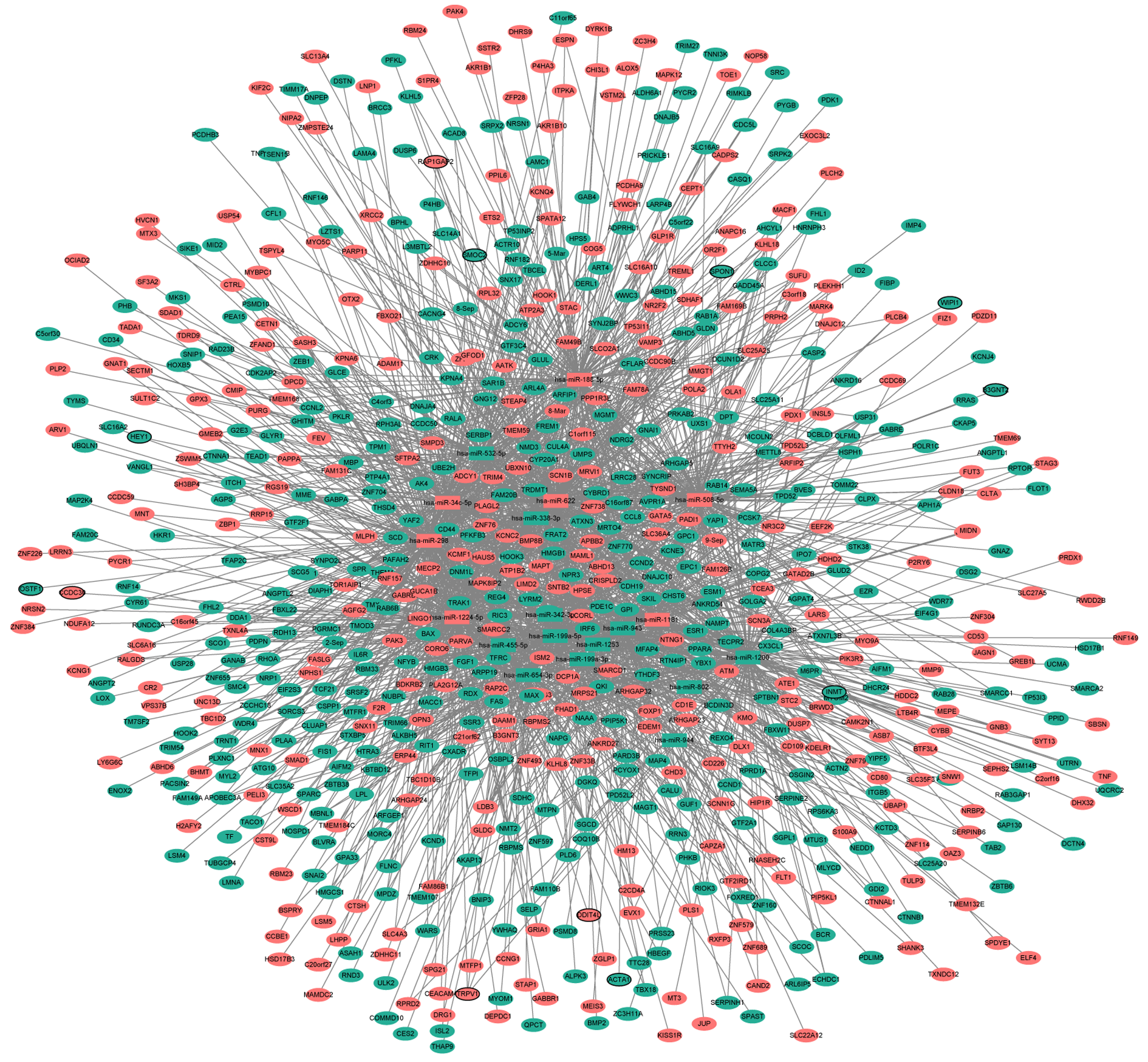

Supplement: Supplementary 2 — Supplementary Figure 2: the miRNA-mRNA network in AF. Rectangle and ellipse represent miRNAs and mRNA, respectively. Red and green colors represent upregulation and downregulation, respectively. [file 8037273.f2.tif]

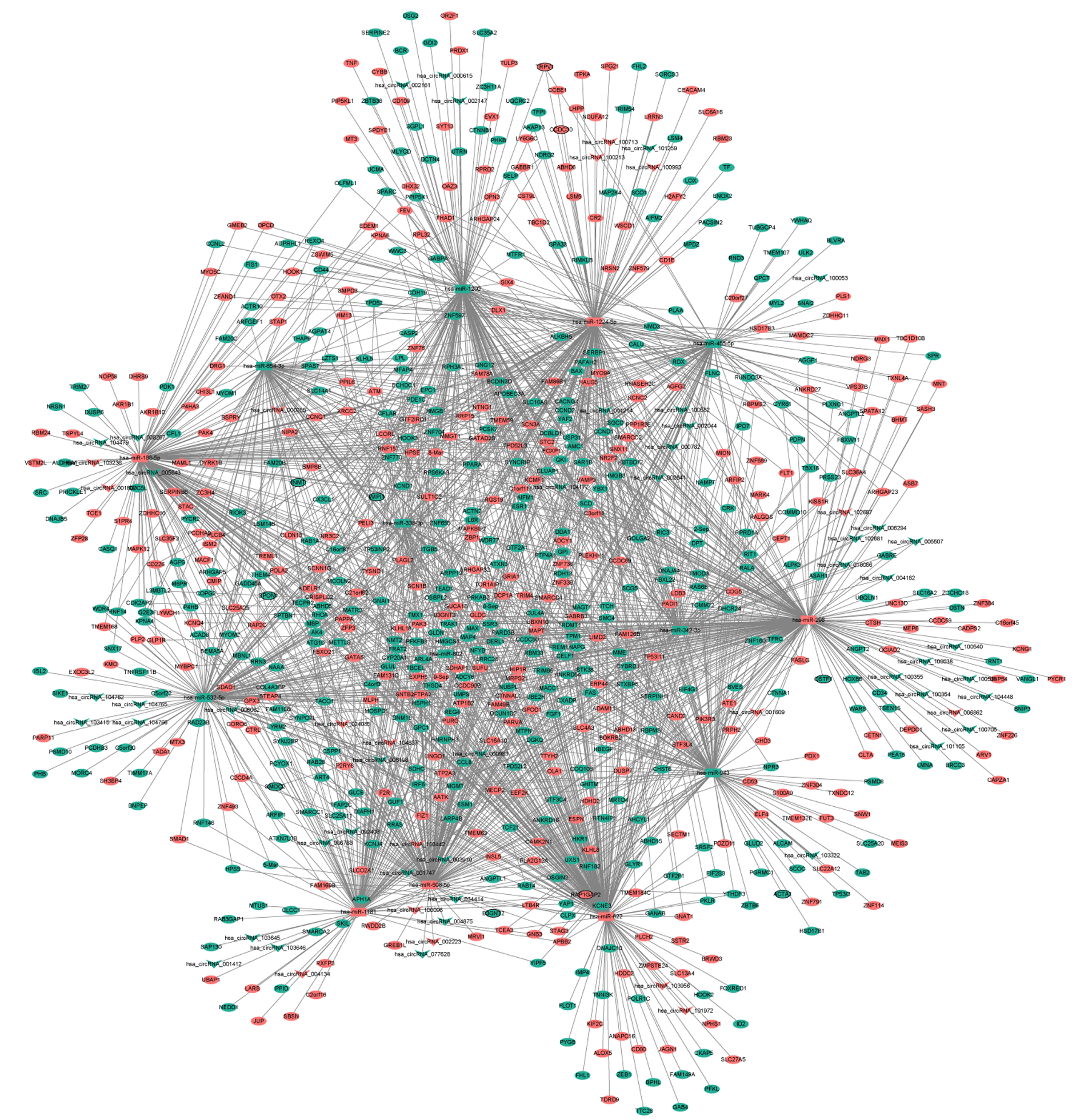

Supplement: Supplementary 3 — Supplementary Figure 3: ceRNA (DEcircRNA-DEmiRNA-DEmRNA) regulatory network. The trigonal nodes, rectangle nodes, and elliptical nodes indicate DEcircRNAs, DEmiRNAs, and DEmRNAs, respectively. Red and green colors represent upregulation and downregulation, respectively. Nodes with the black border were DEcircRNA/DEmiRNA/DEmRNA derived from the top 10 upregulated and downregulated DEcircRNA/DEmiRNA/DEmRNA in AF. [file 8037273.f3.tif]

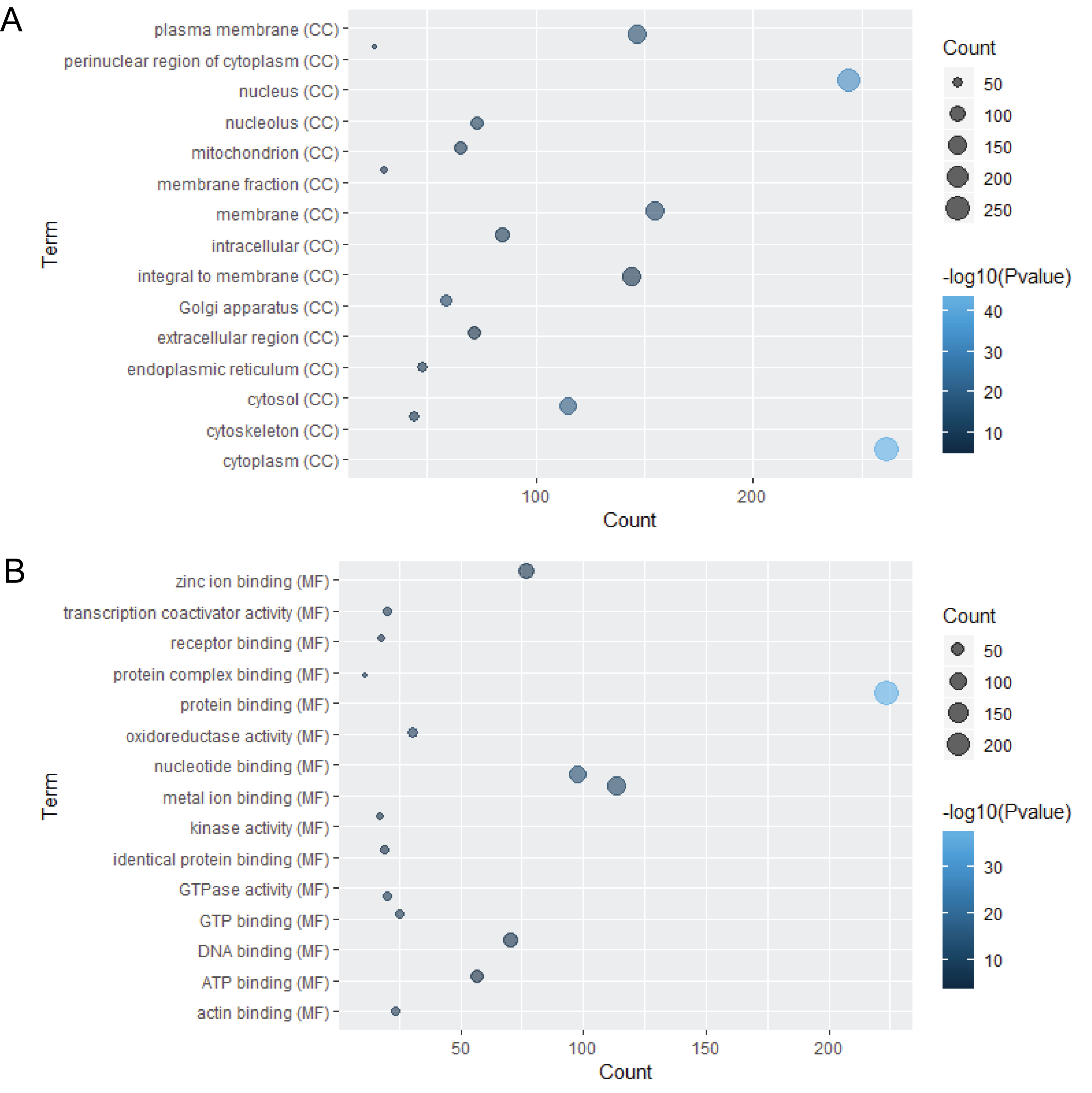

Supplement: Supplementary 4 — Supplementary Figure 4: significantly enriched cytological components and molecular functions of differentially expressed mRNAs in ceRNA regulatory network. (A) CC, cytological components; (B) MF, molecular functions. The x-axis shows counts of host genes enriched in cytological components or molecular functions, and the y-axis shows biological processes or molecular functions. [file 8037273.f4.tif]
