# Supplementary material for: Identification of Circular RNA-MicroRNA-Messenger RNA Regulatory Network in Atrial Fibrillation by Integrated Analysis
Source: Biomed Res Int. 2020 Sep 29;2020:8037273. doi: 10.1155/2020/8037273 (PMC7545447; doi:10.1155/2020/8037273)
Supplement: Supplementary 5 — Supplementary Table 2: the clinical information of AF patients and normal individuals in the qRT-PCR. [file 8037273.f5.doc]

**Supplementary Table 2 The clinical information of AF patients and normal individuals in the qRT-PCR**

| **Group** | **Gender** | **Age** | **BMI** | **Resting heart rate (time/minute)** | **Frequency of AF** | **Smoking history** | **Hypertension** | **Diabetes** | **Dyslipidemia** | **Stroke** | **Serum creatinine (umol/L)** | **Hemoglobin (g/L)** |
| --- | --- | --- | --- | --- | --- | --- | --- | --- | --- | --- | --- | --- |
| AF | Male | 55 | 27.1 | 85 | Continuous | No | Grade 2 | No | Yes | No | 70.5 | 139 |
| AF | Female | 61 | 33.9 | 80 | Continuous | No | No | Yes | Yes | Yes | 75 | 100 |
| AF | Male | 67 | 25.7 | 55 | Continuous | No | Grade 1 | No | Yes | No | 85.5 | 147 |
| AF | Female | 48 | 25.1 | 91 | Continuous | No | No | No | Yes | No | 91 | 88 |
| AF | Female | 32 | 22.2 | 127 | Continuous | No | No | No | Yes | No | 83.1 | 135 |
| AF | Male | 75 | 23.5 | 92 | Continuous | Yes | Grade 3 | No | Yes | No | 73.3 | 175 |
| AF | Male | 64 | 24.7 | 76 | Continuous | No | No | No | Yes | Yes | 79.4 | 154 |
| AF | Male | 75 | 23.5 | 74 | Continuous | Yes | Grade 1 | No | Yes | No | 78.8 | 137 |
| AF | Female | 69 | 32.42 | 120 | Continuous | No | Grade 2 | Yes | Yes | No | 51.3 | 92 |
| AF | Male | 55 | 20.9 | 87 | Continuous | No | No | No | Yes | No | 100.3 | 147 |
| NC | Female | 25 | 19.6 | 76 | No | No | No | No | No | No | 71.3 | 112 |
| NC | Female | 34 | 21.7 | 68 | No | No | No | No | No | No | 44.7 | 128 |
| NC | Female | 24 | 20.8 | 88 | No | No | No | No | No | No | 66.9 | 139 |
| NC | Female | 30 | 20.4 | 67 | No | No | No | No | No | No | 56.2 | 124 |
| NC | Female | 30 | 19.3 | 73 | No | No | No | No | No | No | 88.5 | 119 |
| NC | Female | 27 | 19.8 | 79 | No | No | No | No | No | No | 73.9 | 140 |
| NC | Female | 27 | 22.1 | 82 | No | No | No | No | No | No | 62.4 | 127 |
| NC | Male | 30 | 24.7 | 69 | No | No | No | No | No | No | 60.7 | 132 |
| NC | Male | 32 | 25.3 | 71 | No | No | No | No | No | No | 70.2 | 138 |
| NC | Female | 26 | 21.1 | 77 | No | No | No | No | No | No | 71.3 | 129 |

BMI: body mass index; AF: atrial fibrillation; NC: normal controls
