# Supplementary material for: Identification of Circular RNA-MicroRNA-Messenger RNA Regulatory Network in Atrial Fibrillation by Integrated Analysis
Source: Biomed Res Int. 2020 Sep 29;2020:8037273. doi: 10.1155/2020/8037273 (PMC7545447; doi:10.1155/2020/8037273)
Supplement: Supplementary 6 — Supplementary Table 1: selected circRNA, miRNA, and mRNA datasets. [file 8037273.f6.doc]

**Supplementary Table 1 Selected circRNA, miRNA and mRNA datasets**

| **GEO accession** | **Author** | **Platform** | **Samples (NC: AF)** | **Year** | **Tissue** |
| --- | --- | --- | --- | --- | --- |
| GSE129409 | Ban Liu | GPL21825 074301 Arraystar Human CircRNA microarray V2 | 3：3 | 2019 | Heart |
| GSE68475 | Masaki Morishima | GPL15018 Agilent-031181 Unrestricted_Human_miRNA_V16.0_Microarray 030840 (Feature Number version) | 11：10 | 2017 | Heart |
| GSE70887 | Susana Canon | GPL19546 Agilent-021827 Human miRNA Microarray [miRBase release 17.0 miRNA ID version] | 2：4 | 2015 | Heart |
| GSE31821 | emmanuelle meugnier | GPL570 [HG-U133_Plus_2] Affymetrix Human Genome U133 Plus 2.0 Array | 2：4 | 2018 | Heart |

NC: normal controls; AF: atrial fibrillation.
